# Validation of ultrasonographic muscle thickness measurements as compared to the gold standard of computed tomography in dogs

**DOI:** 10.7717/peerj.2926

**Published:** 2017-01-25

**Authors:** Lindsey E. Bullen, Maria G. Evola, Emily H. Griffith, Gabriela S. Seiler, Korinn E. Saker

**Affiliations:** 1 College of Veterinary Medicine: Department of Molecular Biomedical Sciences, North Carolina State University, Raleigh, NC, United States; 2 College of Sciences: Department of Statistics, North Carolina State University, Raleigh, NC, United States

**Keywords:** Nutrition, Muscle loss, Ultrasound, Computed tomography, Canine, Veterinary, Dog, Muscle thickness, Monitoring, DEXA

## Abstract

**Objective:**

The objective was to quantitatively evaluate the validity of ultrasonographic (US) muscle measurements as compared to the gold standard of computed tomography (CT) in the canine.

**Design:**

This was a prospective study.

**Population:**

Twenty-five, client-owned dogs scheduled for CT as part of a diagnostic work-up for the management of their primary disease process were included.

**Materials and Methods:**

Specific appendicular (*cubital flexors and extensors, coxofemoral flexors and extensors*) and axial (*temporalis, supraspinatus, infraspinatus, lumbar epaxials*) muscle groups were selected for quantitative measure based on CT planning and patient position. Prior to CT scan, the skin over the muscle sites was shaved and marked with a permanent marker. Patient body position was determined based on the patient’s CT plan; positioning was consistent between CT and US imaging. To ensure identical imaging position for both CT and US measurements, radio-opaque fiducial markers were placed directly over the skin marks once the dog was positioned. Quantitative measurements (cm) for both lean muscle mass (LMM) and subcutaneous adipose (SQA) were recorded. Statistical comparisons between CT and US values were done separately for each site and type.

**Results:**

Muscle groups and associated SQA measured by US and CT were not statistically different based on an adjusted *p*-value using Bonferroni’s correction (*p* < 0.0031). In addition, all LMM and SQA sites had good reliability and agreement (Cronbach’s *α* = 0.8 − 1.0) between the two metrics, excluding the coxofemoral extensor muscle group (Cronbach’s *α* = 0.73232). Linear regression analysis of muscle measures indicated close agreement (slope range 0.93–1.09) and minimal bias of variation (intercept range 0.05–0.11) between CT versus US modalities, with the exception of the coxofemoral extensor muscle. Similarly, SQA CT and US measures indicated close agreement with the slope range of 0.88–1.02 and minimal bias of variation with an intercept range of 0.021–0.098, excluding the cubital flexor and extensor groups. Additionally, the *R*^2^ values for these remaining LMM and SQA sites are reported as >0.897 for LLM and >0.8289 for SQA.

**Conclusions:**

Ultrasound imaging of selected appendicular and axial muscle groups in dogs can provide comparable assessment of muscle thickness to the current gold standard, CT. In consideration of both statistical reliability to CT and cage-side accessibility, the *temporalis, supraspinatus, infraspinatus,* and *lumbar epaxial* LMM sites are considered the most useful targets for US LMM assessment in the canine. Our findings support the potential utility of US as a clinical tool in veterinary medicine to assess LMM status in patients. Additional studies are indicated to develop standardized protocols of its use in a cage-side setting and to elucidate the benefit of this modality, in conjunction with nutritional interventions, to manage body LLM stores in compromised patients.

## Introduction

Skeletal or lean muscle mass (LMM) loss from chronic wasting diseases, disuse, or traumatic injury has a profound impact on the patients overall physical and metabolic state. Lean muscle mass is the largest pool of protein in the body and is essential for appropriate immune function, inflammatory response, glucose disposal, protein synthesis, and mobility; this is especially relevant during times of illness. Given that LMM is a direct reflection of total body protein stores, loss of LMM then represents a depletion of total body protein and thus loss of mobilizable protein reserves (Tsai, 2012; Moisey et al., 2013; Mourtzakis & Wischmeyer, 2014). Singularly or in combination, the consequences of total body LMM loss can negatively impact mortality, as well as length of ICU stay and overall hospitalization (Lieffers et al., 2012; Moisey et al., 2013; Mourtzakis & Wischmeyer, 2014).

Muscle wasting occurs early, within the first seven days of a disease course, and rapidly as LMM loss has been quantitated by day 2–3 of hospitalization (Campbell et al., 1995; Gruther et al., 2008; Tillquist et al., 2014). Studies report that LMM stores decline in hospitalized patients, especially when compounded by malnourishment, sarcopenia, illness, aging, or sepsis. This decline adversely impacts diaphragmatic and gastric function, length of hospital stay, medication-related toxicities, incidence of nosocomial infections, and ventilator time (Cosqueric et al., 2006; Griffiths & Hall, 2009; Lieffers et al., 2012; Tsai, 2012; Di Sebastiano et al., 2013; Moisey et al., 2013). The early detection and sequential monitoring of LMM changes in at-risk patient populations may mitigate the above mentioned biochemical and metabolic complications, hasten recovery rates and improve their quality of life by allowing for early and appropriate nutritional and medical interventions. While nutritional interventions may not completely reverse muscle loss and subsequent metabolic alterations, targeted nutrition has been shown to slow and delay the muscle loss process (Evans, 2010).

In human medicine, three modalities traditionally are used to analyze body composition: computed tomography (CT), magnetic resonance imaging (MRI), and dual energy x-ray  absorptiometry (DEXA). CT and MRI are considered to be the gold standards for quantitative assessment and analysis of body composition. These modalities can clearly delineate between skin, SQA, lean muscle, and bone to provide a valid, accurate, and reliable estimate of whole body tissue composition. There are several shortcomings of CT and MRI hindering the routine use in a clinical setting including: they are quite costly as a BC assessment tool; respiration can cause image interference; these instruments are not mobile enough for bedside accessibility; CT requires a high dose of ionizing radiation and repeated routine measurements could raise patient safety concerns; and MRI is often limited to highly specialized settings and is thus unavailable for routine use. Dual energy x-ray absorptiometry (DEXA), an alternative non-invasive method of tracking BC, has the limitations of being a stationary instrument and having wide variability in hardware and software between manufacturers. Additionally, DEXA measures can be influenced by hydration status and excess body thickness which can disproportionately shift energy photon levels leading to an underestimation of adipose and LMM compartments (Prado & Heymsfield, 2014). Historically, bedside-accessible tools to assess malnutrition and morbidity/mortality risk in humans has been determined using body mass index (BMI), serum albumin, and anthropometric measures of skin fold and limb circumference (Moisey et al., 2013). These measures are assumed, directly or indirectly, to be reflective of the patients LMM and/or body protein status. Although they are easily obtained and relatively low cost, the true value of these measures to assess either LMM or protein status has been questioned as to their accuracy and reliability (Prado & Heymsfield, 2014). BMI measures do not distinguish between adipose, muscle and water. Serum albumin can be significantly affected by changes in intravascular volume, organ dysfunction, cutaneous wounds, inflammation, and sequestration or third spacing. Fluid retention and subcutaneous edema confound measures obtained by skin fold analysis, especially in the critically ill and/or elderly patient (Gough, 2007; Kuzuya et al., 2007; Moisey et al., 2013). Campbell et al. (1995) sought to identify a clinically applicable marker to assess body composition independent of hydration status. They compared muscle measures via skin fold thickness, DEXA and ultrasound (US) at three sites (biceps, anterior forearm, and anterior thigh) between healthy and critically ill human subjects suffering from multiple organ failure (MOF) and exhibiting edema. Correlation coefficients of the three US measurements with lean tissue mass from DEXA were significantly stronger than those derived from skin-fold thickness, especially in the MOF subjects, as edematous fluid is retained in the SQA not the muscle body. Although US is well-established as a method of monitoring muscle mass changes (Prado & Heymsfield, 2014), Campbell et al. (1995) further identified it as a reliable modality for LMM assessment in critically ill, hospitalized patients with variations in hydration status, which is a major limitation among other commonly utilized assessment tools.

Although investigators have further verified utility of US as a quantitative assessment of LMM in the bed-bound patient and the correlation of US measures with DEXA-derived measures, there is only one published study evaluating US against CT, the reported gold standard for BC assessment (Thomaes et al., 2012; Puthucheary et al., 2013; Takai et al., 2013; Mourtzakis & Wischmeyer, 2014; Tillquist et al., 2014). To the authors’ knowledge, US quantification and longitudinal monitoring of LMM has not yet been validated in non-human species.

Similar to humans, veterinary patients suffer with critical acute and chronic disease states which can rapidly drain protein reserves. Confounding factors such as life-stage, reproductive status, and current nutritional status present added complexity and increased risk for malnutrition during their recovery period. Subsequently, accurate assessment of changes in LMM would enable optimization of targeted therapies (nutritional, medical, and pharmacological) in a timely manner to maximize overall patient management in this population as well as in human patients. The validation of US muscle measurements as an accurate, non-invasive, inexpensive, low-health risk, and easily operated modality for cage-side (clinical) use with companion animals will be a first step in addressing the negative ramifications of malnutrition associated with loss of LMM in veterinary patients (Zaiman et al., 2014). It will likewise serve as an invaluable tool to assess the veterinary patient with neurodegenerative and musculoskeletal disease(s), allowing for targeted nutritional and rehabilitative protocols. Therefore, the overall goal for this study was to *validate the use of cage-side quantitative ultrasound (US) assessment of LMM against the reported gold standard of CT measure in reliably accessible muscle sites in the canine.*

## Materials and Methods

Twenty-five dogs undergoing CT as part of their critical illness diagnostic work-up at a teaching hospital in Raleigh, North Carolina were identified for, and enrolled in this study. LMM regions identified for investigation included the: temporalis, supraspinatus, infraspinatus, cubital flexor and extensor groups, lumbar epaxial, and coxofemoral extensor and flexor groups. For each patient, LMM sites were only investigated if their preordained CT plan included those regions. However, due to patient positioning and CT plan, the coxofemoral flexor group did not have an adequate sample size (*n* = 1) to be statistically  evaluated.

Prior to CT, palpation was used to identify the muscle regions of interest; specific anatomic landmarks were not used to standardize investigation location across patients as each patient and site was its own control. The hair was shaved and skin was marked with permanent ink over the LLM regions being investigated. A radio-opaque fiducial marker (PinPoint for Image Registration; Beekley Medical, Bristol, CT, USA) was then placed directly over the marked skin on the specified LMM sites to ensure identical measurement positions via CT and US. Cross-sectional images of 1 mm for each LMM site were obtained by a helical 64 slice CT (Siemens Somatom 64 CT Scanner; Siemens, Forchhiem, Germany). These images were later reconstructed, viewed, and analyzed in MergePacs™ Workstation’s multiplanar reconstruction viewport (MergePacs Workstation, Version 6.5; Merge Healthcare, Hartland, WI, USA). Computed tomography images were reconstructed in order to mimic the US plane of insonation. Additionally, all CT images were viewed in a soft tissue window (width = 400; level = 40). The subcutaneous adipose (SQA) tissue to muscle interface and LMM to bone interface were identified. The distance between the contact point of the fiducial marker to skin and SQA to LMM interface was delineated as SQA. Correspondingly, the distance between SQA to LMM interface and LMM to bone interface was delineated as LMM.

**Figure 1:**
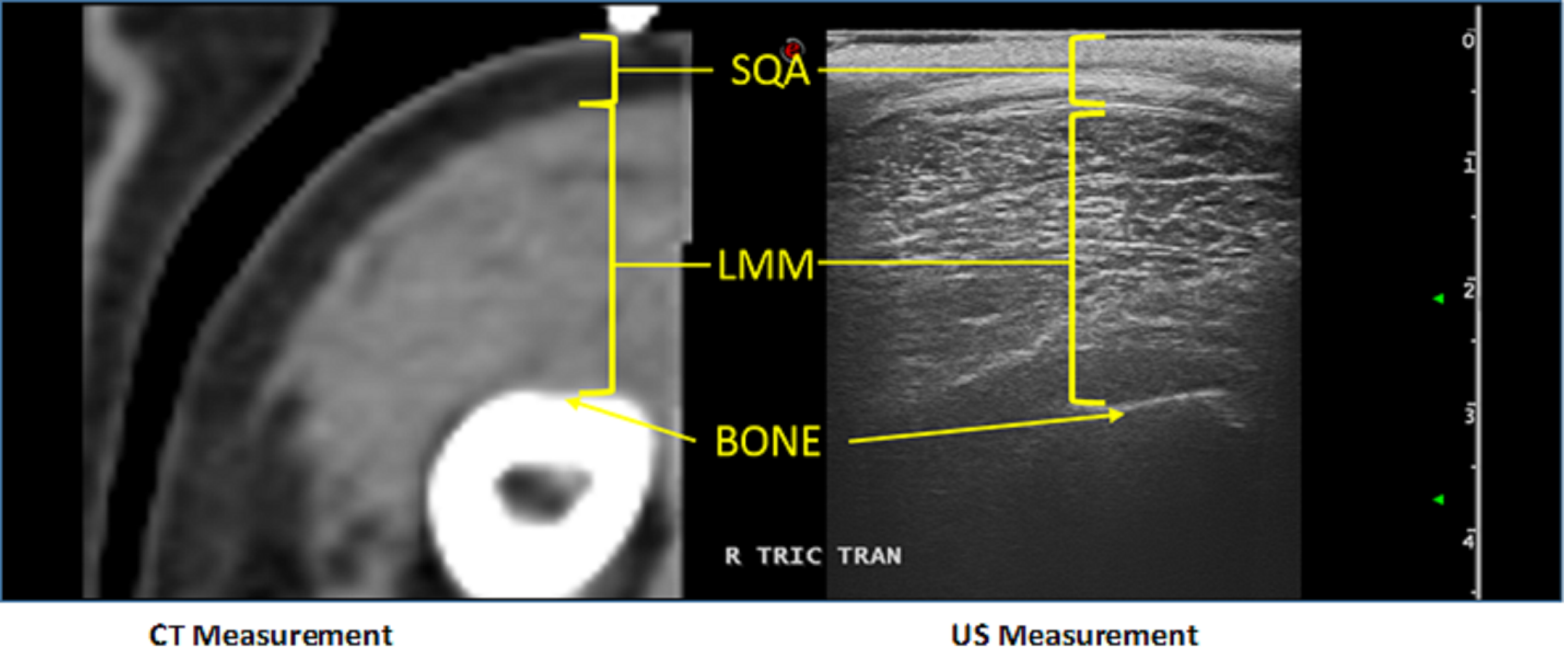
Example of the comparison between US and CT measurements for a canine patient’s cubital extensor muscle group.

Immediately following CT imaging, the fiducial marker was removed and US images were acquired using B-mode ultrasound (Esaote MyLab™ Twice; Esaote North America, Indianapolis, IN, USA) and a linear transducer (LA 523 Variable-band linear array; Esaote North America, Indianapolis, IN, USA) with a 4 MHz–13 MHz scanning frequency. Water-soluble ultrasound transmission gel (Aquasonic 100 Ultrasound Transmission Gel; Parker Laboratories, INC., Fairfield, NJ, USA) was used to coat the probe head to provide optimal acoustic contact while light manual pressure was used to minimize SQA and LMM compression, so as not to affect ultrasound measurements. As tangential and oblique images can artificially increase muscle thickness measurements, the transducer was centered directly over the previously marked skin, perpendicular to the LMM area of investigation. Additionally, both transverse and longitudinal images were captured. Measurements were taken using electronic calipers on a still image (Fig. 1). Both CT and US were performed by clinicians with advanced veterinary radiologic imaging training (board-certified radiologist (DACVR/ DECVDI) or senior radiology resident).

Patient position was predetermined based on disease process and CT plan. However, patients were not moved nor their positions altered between CT and US to ensure identical muscle positioning during investigation with the two modalities. Units of measure for US and CT to quantitate muscle thickness were identical (to the nearest 100th mm), allowing for straightforward correlation between imaging modalities. Anesthesia protocol was identified, instituted, and monitored for each individual dog by an Anesthesiology clinician. Following US, patients were managed by their primary clinician(s) for their presenting complaint and/or continued treatment. All dogs enrolled in the study were treated humanely in accordance with the North Carolina State University Institutional Animal Care and Use Committee guidelines, which provided full approval for this study. (#14-186-O).

### Statistical analysis

All statistical analyses were performed in standard statistical software (SAS, Version 9.4; Cary, NC, USA). All comparisons were done separately for each site and type. Paired *t*-tests were used to check for mean differences between US and CT measurements within each animal and each site. Using Bonferroni’s correction to adjust significance level, all *p*-values were compared to a cutoff value of 0.0031. Cronbach’s alpha was calculated for each type and location to assess agreement between the two types of measurements, with values less than 0.8 having poor agreement. Linear regression models were fit to compare the size and direction of any differences between the US and CT measurements.

### Results

**Figure 2:**
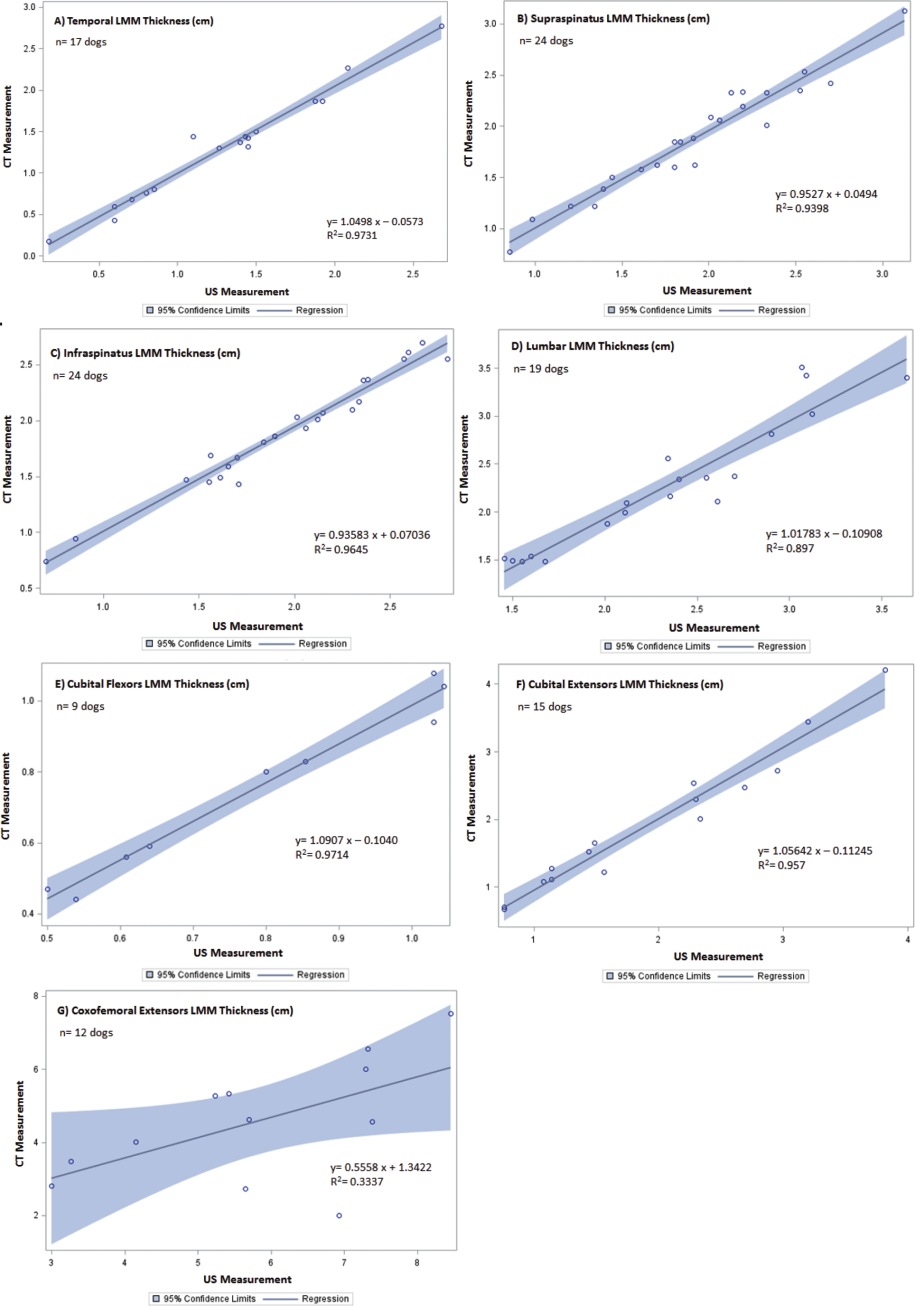
Regression between CT and US LMM measurements of canine patients included in the validation of US vs. CT. The line of regression (solid), and 95% confidence limits (shaded) are given for (A) temporal LMM, (B) supraspinatus LMM, (C) infraspinatus LMM, (D) lumbar LMM, (E) cubital flexor LMM, (F) cubital extensor LMM, and (G) coxofemoral extensor LMM.

**Figure 3:**
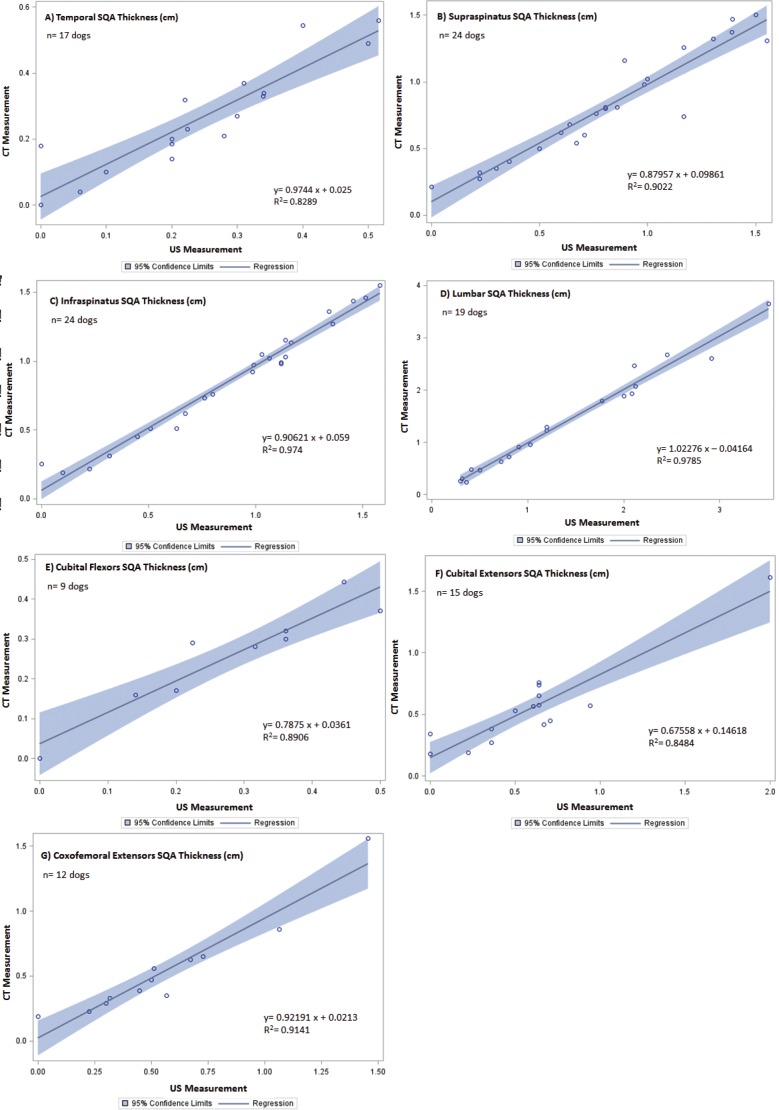
Regression between CT and US SQA measurements of canine patients included in the validation of US vs. CT. The line of regression (solid), and 95% confidence limits (shaded) are given for (A) temporal SQA, (B) supraspinatus SQA, (C) infraspinatus SQA, (D) lumbar SQA, (E) cubital flexor SQA, (F) cubital extensor SQA, and (G) coxofemoral extensor SQA.

Of the twenty-five dogs enrolled, the study population included both mixed breed and pure breed dogs, with equal representation of males and females ranging in age, body weight, body condition and muscle mass index. Demographics of the patients are summarized in Table 1. The CT and US measurements of each muscle region were evaluated. Slices of the CT scan were reconstructed to best compare LMM and SQA measures between the two modalities; an example is shown in Fig. 1. Although 25 dogs were available for the combination CT and US measurements, there were inadequate LMM and SQA coxofemoral flexor group readings available to allow for statistical evaluation. However, for the remaining LMM groups there were no statistically significant differences (*P* < 0.0031 between CT and US measurements) (Table 2). In addition, these LMM sites had good reliability and agreement between US and CT (Cronbach’s *α* between 0.8 and 1.0) (Table 2) with the exception of the coxofemoral extensor group (0.73232). The SQA site measurements associated with specific LMM groups were likewise not statistically different between CT and US; and all SQA sites including coxofemoral extensors, had good reliability and agreement between CT and US (Table 3). Regression analysis was implemented on both LMM and SQA data to determine the extent of correlation between CT and US. With the exception of the coxofemoral extensor LMM (slope = 0.5558; intercept = 1.3422; *R*^2^ = 0.3337) and cubital extensor SQA (slope = 0.67558; intercept = 0.14618; *R*^2^ = 0.8484), each site’s analysis denoted the slope and intercept were close to 1 and 0, respectively, indicating close agreement and minimal bias. Additionally, the *R*^2^ values for these remaining LMM and SQA sites are reported as >0.897 for LLM and >0.8289 for SQA (Figs. 2 and 3).

**Table 1:** Demographics/physical characteristics of canine patients included in the validation of US vs. CT muscle thickness measurements.

Variables	*N*	Median	Mean	SD	Min	Max
Female intact	0					
Female spayed	13					
Male intact	3					
Male castrated	9					
Age (y)	25	10	9.1	3.08	1.5	13
Weight (kg)	25	26.3	23.34	12.07	4.95	45.8
BCS (1–9)	25	5	5.38	1.59	3	9
MMI (0–3)	25	2	2.4	0.5	2	3

**Table 2:** Statistical comparison of LMM sites of canine patients included in the validation of US vs. CT muscle thickness measurements.

Site	*N*	Mean paired difference (cm)	95% CI	^*^P-value	^a^Cronbach’s *α*
Temporalis	17	0.007	−0.066–0.052	0.8114	0.99319
Supraspinatus	24	0.041	−0.016–0.098	0.1481	0.98447
Infraspinatus	24	0.055	0.012–0.097	0.0141	0.99096
Cubital Extensors	15	0.004	−0.117–0.124	0.9509	0.98901
Cubital Flexors	9	0.033	−0.002–0.068	0.0621	0.99275
Lumbar Epaxials	19	0.067	−0.039–0.173	0.1989	0.97284
Coxofemoral Extensors	12	1.272	0.251–2.232	0.0186	0.73232

**Notes.**

* *P* < 0.0031 is considered statistically significant, having been adjusted with Bonferroni’s correction.

a Values <0.8 ≠ reliable or agreeable. 0.8–1.0 have both good reliability and agreement.

**Table 3:** Statistical comparison of SQA sites of canine patients included in the validation of US vs. CT muscle thickness measurements.

Site	*N*	Mean paired difference (cm)	95% CI	^*^P-value	^a^*Cronbach’sα*
Temporalis	17	0.019	−0.054–0.016	0.2706	0.95312
Supraspinatus	24	0.001	−0.056–0.058	0.9774	0.97426
Infraspinatus	24	0.025	−0.008–0.058	0.1358	0.99341
Cubital extensors	15	0.047	−0.065–0.159	0.3842	0.95892
Cubital Flexors	9	0.024	−0.018–0.066	0.2231	0.97106
Lumbar epaxials	19	0.010	−0.061–0.080	0.7771	0.99458
Coxofemoral extensors	12	0.023	−0.050–0.095	0.5030	0.97754

**Notes.**

* *P* < 0.0031 is considered statistically significant, having been adjusted with Bonferroni’s correction.

a Values <0.8 ≠ reliable or agreeable. 0.8–1.0 have both good reliability and agreement.

## Discussion

Appropriate LMM is essential for numerous biologic functions. The combination of ongoing disease processes, limited mobility during illness and recovery, and inadequate nutritional support results in rapidly occurring loss of LMM. This undesired loss of LMM can result in severely detrimental ramifications for the patient (Lightfoot, McArdle & Griffiths, 2009; Tsai, 2012; Mourtzakis & Wischmeyer, 2014). Human medicine has documented delayed wound healing and recovery, impaired immune function, and metabolic derangements all associated with loss of LMM (Lieffers et al., 2012; Moisey et al., 2013; Mourtzakis & Wischmeyer, 2014). US-guided muscle assessment could be beneficial to help mitigate complications associated with muscle loss by providing earlier medical and nutritional interventions (Evans, 2010; Prado & Heymsfield, 2014).

While US has been validated and used successfully in human medicine for over 20 years (Mourtzakis & Wischmeyer, 2014), to our knowledge, this is the first study evaluating and validating the use of US to assess LMM in the veterinary patient. This prospective study demonstrates that the use of US to quantify specified LMM groups is appropriate for use in canine patients. While validation of ultrasonographic evaluation of LMM was the main objective, the US and CT measurements included adipose tissue in addition to LMM. Regardless of patient BCS and MMI, there were no statistically significant differences between CT and US measurements for both LMM and SQA at each site evaluated. Although not identified as a specific objective, determining which muscle groups are both accurate and easily accessible are important for clinical use. Despite the strong correlation between CT and US, all evaluated sites are not practical for cage-side use. It was the consensus of all involved radiologists that the sites associated with the appendicular skeleton were by far the most difficult to technically and physically access and image. While the appendicular musculature is assessed routinely in human medicine, the anatomic conformation of quadrupeds makes US assessment of these sites challenging (Campbell et al., 1995; Puthucheary et al., 2013; Mourtzakis & Wischmeyer, 2014; Tillquist et al., 2014). In addition, critically ill veterinary patients are typically rested in alternating lateral recumbency while humans are in a supine position (Griffiths & Hall, 2009). This difference contributes to the challenge of assessing the appendicular musculature of the veterinary patient. The sites most easily accessed and imaged by the ultrasonographers included those not influenced by our patients’ quadruped nature: the temporalis, the supraspinatus, the infraspinatus, and the lumbar epaxials.

There are several limitations of this study to consider. While preliminary data and power analyses indicated a minimum of *n* = 24 for each site, due to CT planning and patient positioning, this target number was only achieved on two sites, the supra- and infraspinatus. Statistical significance was likely affected by the smaller than desired sample size for the remaining site. In order to determine if the effect is positive or negative, additional measurements would be required. In addition, the US probe had difficulty imaging structures at depths greater than 6 cm. It would be extremely challenging if not impossible to evaluate large patients whose LMM have a thickness greater than 6 cm with the ultrasound probe used in this study (4 MHz–13 MHz). In that situation, a different ultrasound probe with lower scanning frequencies could be used to achieve ultrasound images at deeper depths. Alternatively, it may be more feasible to use a combination of US measured SQA and anthropometric measures to monitor changes. Another limitation is that this study only included canine patients. While inclusion of other species was beyond the scope of this study, canine patients as small as 4.95 kg were included with results comparable to those of larger patients. It is therefore reasonable to extrapolate and infer comparable modality correlation due to the similarity of anatomy between our veterinary canids and felids (Coulson & Lewis, 2002).

US may be a useful modality in assessing and identifying patients at risk for rapid muscle wasting, which can be both compounded by and a product of states of malnutrition, immobility, and many disease states. In order to maximize the full potential of cage-side US use, additional studies are indicated. Future goals include: longitudinal tracking of muscle mass changes in both critically ill and rehabilitation patients; and evaluation of the intra- and inter-rater variability between ultrasonographers and to develop a training guide for potential users. Ultimately, further investigation is warranted to elucidate the relationship between LMM fluctuations, nutritional status, and protein homeostasis with the end goal being to assess response to targeted nutritional, medical, and rehabilitation therapies aimed at countering LMM loss. Notwithstanding the above-mentioned challenges and limitations, this study supports the use of US to initially assess and serially monitor changes in LMM and SQA during hospitalization, recovery, and/or rehabilitation.

##  Supplemental Information

10.7717/peerj.2926/supp-1 Data S1 Raw Data

## Abbreviations

BC: Body composition
BCS: Body condition score
BMI: Body mass index
cm: Centimeter
CT: Computed tomography
DEXA: Dual energy x-ray absorptiometry
kg: Kilogram
LMM: Lean muscle mass
MHz: Megahertz
mm: Millimeter
MMI: Muscle mass index
MRI: Magnetic resonance imaging
SQA: Subcutaneous adipose
US: Ultrasonographic

## Additional Information and Declarations

### Data Availability

The following information was supplied regarding data availability: The raw data has been supplied as a Data S1.
